# Malignant gliomas with *H3F3A* G34R mutation or *MYCN* amplification in pediatric patients with Li Fraumeni syndrome

**DOI:** 10.1007/s00401-021-02346-8

**Published:** 2021-07-15

**Authors:** Melanie Schoof, Uwe Kordes, Alexander E. Volk, Sina Al-Kershi, Catena Kresbach, Ulrich Schüller

**Affiliations:** 1 Department of Pediatric Hematology and Oncology, University Medical Center Hamburg-Eppendorf, Hamburg, Germany; 2 Research Institute Children’s Cancer Center Hamburg, Hamburg, Germany; 3 Institute of Human Genetics, University Medical Center Hamburg-Eppendorf, Hamburg, Germany; 4 Institute of Neuropathology, University Medical Center Hamburg-Eppendorf, Hamburg, Germany

Patients with Li Fraumeni syndrome (LFS) are prone to a variety of cancers including tumors of the central nervous system. In particular, such patients frequently develop choroid plexus carcinomas, SHH medulloblastomas, or IDH-mutated astrocytomas (for a review see [1]). IDH wild type glioblastomas have recently been observed in three LFS patients [2]. However, due to the small number of described cases, both the frequency and the molecular spectrum of such glioblastomas remain less clear. We report on two additional children with LFS, who developed malignant IDH wild type glioma, one of them (case 1) with a diffuse hemispheric glioma, H3 G34-mutant, and one of them (case 2) with a diffuse pediatric-type high grade glioma, H3 and IDH wild type with *MYCN* amplification. The former was a 13-year old boy with a positive family history for LFS and a parieto-temporal left-sided lesion (Fig. 1a–b) that turned out to be a high grade glioma by histology (Fig. 1e). Immunohistochemistry showed very strong nuclear accumulation of p53 in the tumor cell nuclei (Fig. 1f) as well as binding of antibodies against mutant H3.3 G34 (Fig. 1g). Sequencing of the tumor confirmed the somatic *H3F3A* G34R mutation and revealed the pathogenic *TP53* variant R175H (c.524G > A) in tumor and leukocyte derived DNA. Case 2 presented as a 13-year old boy with an occipital lesion in the right hemisphere (Fig. 1c–d). Histology displayed a cell dense and rather undifferentiated tumor (Fig. 1h) with strong nuclear accumulation of p53 (Fig. 1i) and expression of MYCN (Fig. 1j). FISH analysis finally proved a high level amplification of *MYCN* (Fig. 1j, insert), and sequencing detected the *TP53* variant R156G (c.466C > G) that was also present in leukocyte derived DNA. This variant has not been described before. According to ACMG/AMP guidelines, it is classified as a likely pathogenic variant (criteria PM1, PM2, PM5 fulfilled). Both tumors have also been analyzed via global DNA methylation profiling and showed significant matches to the DNA methylation classes of glioblastoma, IDH wild type, H3.3 G34 mutant (case 1) and to the methylation class of glioblastoma, IDH wild type, subclass MYCN (case 2, www.molecularneuropathology.org, classifier version 11b4 [3]). *T*-distributed stochastic neighbor embedding (*t*-SNE) confirmed these results and verified that these cases clustered away from each other in distinct methylation classes (Fig. 1k). Copy number profiles that were inferred from the DNA methylation data showed various chromosomal gains and losses for both cases and focal amplifications at the loci covering *MYCN* and *CDK4* in case 2 (Supplementary Fig. 1, online resource).

**Fig. 1:**
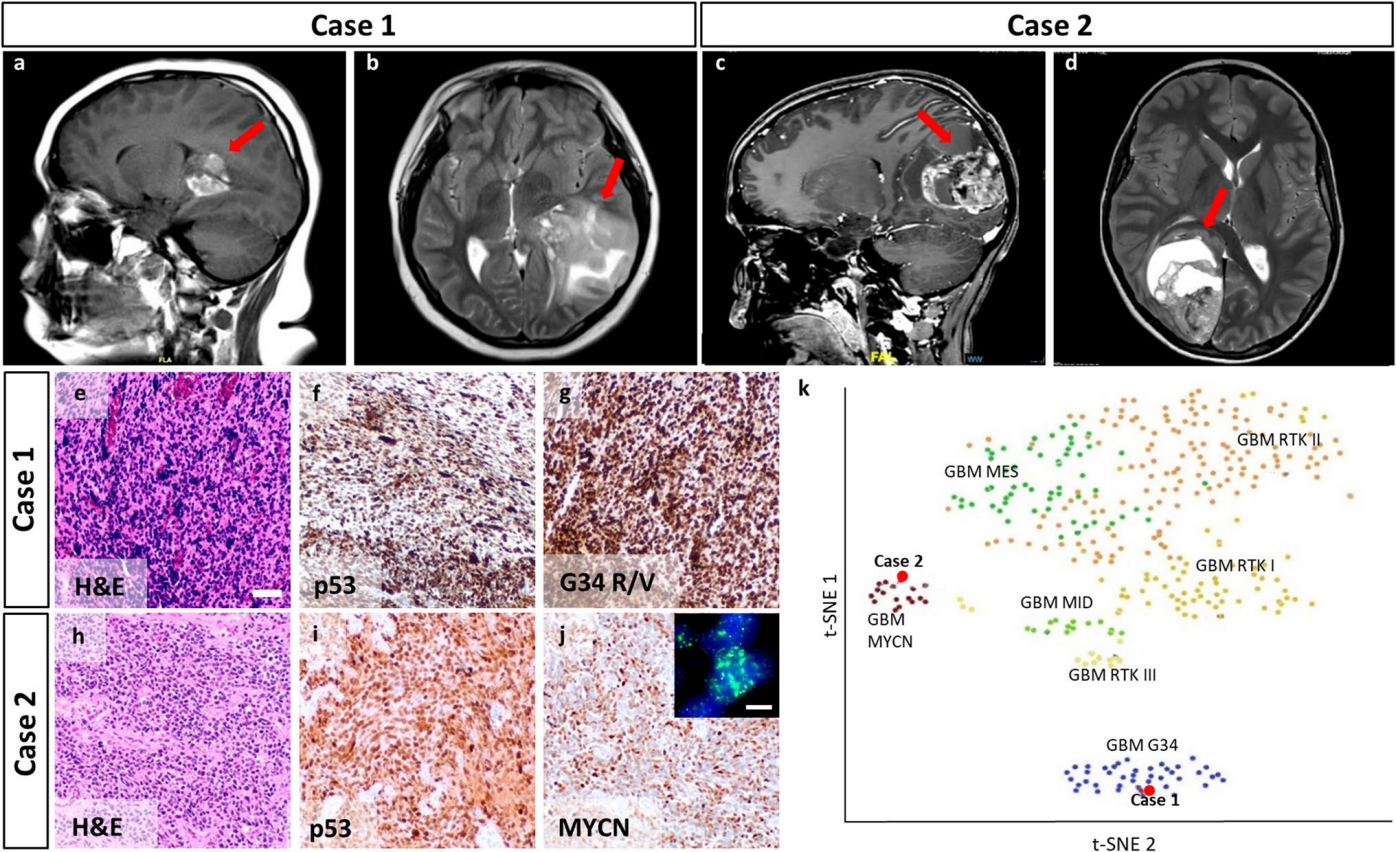
Neuropathology of malignant gliomas occurring in two LFS patients. Magnetic resonance imaging (MRI) of the two cases shows large tumors in the forebrain of both patients (**a**–**d**). H&E histology of case 1 reveals a pleomorphic glial phenotype (**e**), while immunohistochemistry uncovers strong expression of p53 (**f**) and mutant H3.3 as revealed by antibodies detecting tumor cells with *H3F3A* G34 R/V mutations (**g**). Case 2 is characterized by a rather undifferentiated tumor cell morphology (**h**), but similarly strong expression of p53 (**i**). MYCN protein is heavily expressed (**j**) due to a high level amplification of the *MYCN* gene (green signal in inset). *T*-SNE analysis together with a brain tumor reference cohort of glioblastoma (GBM) [3] shows the clustering of case 1 to the DNA methylation classes of glioblastoma, IDH wild type, H3.3 G34 mutant (GBM G34) and of case 2 to the methylation class of glioblastoma, IDH wild type, subclass MYCN (GBM MYCN) (**k**). Scale bar in **e** corresponds to 50 µm in **e–j**. Scale bar in the inset of **j** corresponds to 5 µm

Malignant gliomas with *H3F3A* G34R mutation are well described in the literature with respect to the co-occurrence of *TP53* mutations. A large series of 81 cases, for instance, detected *TP53* mutations in 88% of the tumors [4]. However, it is unclear whether these mutations were of somatic or constitutional (germline) origin. Also, H3.3 G34 mutations have never been specifically looked at or detected in malignant gliomas of LFS patients. Similarly, few reports point to a rather high frequency of *TP53* mutations in malignant pediatric glioma with *MYCN* amplification [5], but the risk of *TP53* constitutional mutations has never been assessed. On the other hand, two cases of IDH wild type gliomas in LFS patients have only recently been described to harbor *MYCN* amplifications [2]. Together, the here presented cases expand the spectrum of brain tumors occurring in patients with LFS. Importantly, these cases underline that patients diagnosed with G34 mutated or MYCN-altered malignant gliomas may carry constitutional *TP53* mutations and should undergo genetic counseling.

## Supplementary Information

Below is the link to the electronic supplementary material.

Supplementary file1 (PPTX 559 KB)

## References

[1] Orr B, Clay M, Pinto E, Kesserwan C (2020). An update on the central nervous system manifestations of Li-Fraumeni syndrome. Acta Neuropathol.

[2] Sloan E, Hilz S, Gupta R, Cadwell C, Ramani B, Hofmann J (2020). Gliomas arising in the setting of Li-Fraumeni syndrome stratify into two molecular subgroups with divergents clinicopathologic features. Acta Neuropathol.

[3] Capper D, Jones DTW, Sill M, Hovestadt V, Schrimpf D, Sturm D (2018). DNA methylation-based classification of central nervous system tumours. Nature.

[4] Korshunov A, Capper D, Reuss D, Schrimpf D, Ryzhova M, Hovestadt V (2016). Histologically distinct neuroepithelial tumors with histone 3 G34 mutation are molecularly similar and comprise a single nosologic entity. Acta Neuropathol.

[5] Korshunov A, Schrimpf D, Ryzhova M, Sturm D, Chavez L, Hovestadt V (2017). H3-/IDH-wild type pediatric glioblastoma is comprised of molecularly and prognostically distinct subtypes with associated oncogenic drivers. Acta Neuropathol.

