# Supplementary material for: Malignant gliomas with H3F3A G34R mutation or MYCN amplification in pediatric patients with Li Fraumeni syndrome
Source: Acta Neuropathol. 2021 Jul 15;142(3):591–3. doi: 10.1007/s00401-021-02346-8 (PMC8357758; doi:10.1007/s00401-021-02346-8)
Supplement: Supplementary file 1 — Supplementary file1 (PPTX 559 KB) [file 401_2021_2346_MOESM1_ESM.pptx]

## Slide 1
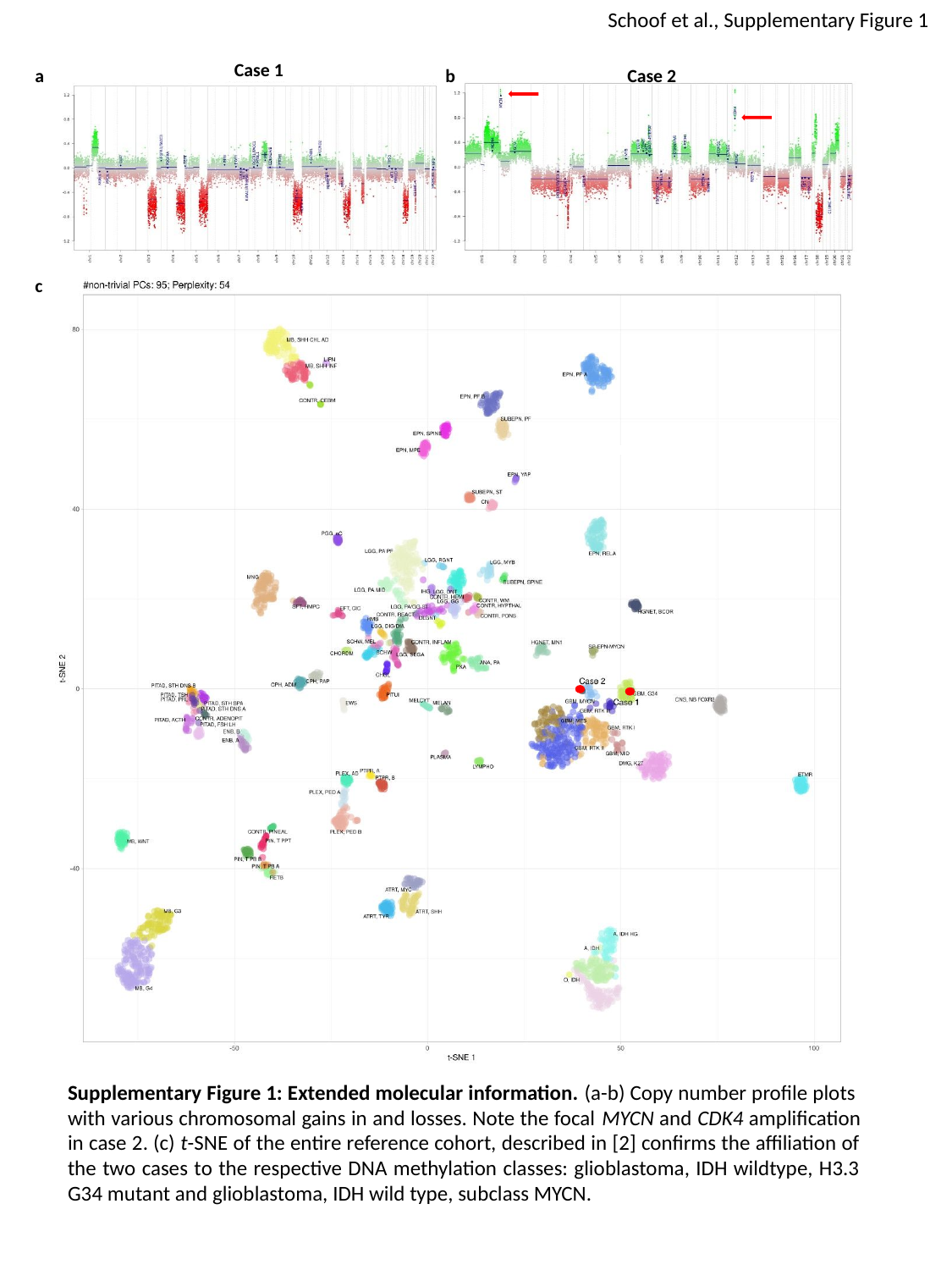

Schoof et al., Supplementary Figure 1
Case 1
a
b
Case 2
c
Supplementary Figure 1: Extended molecular information. (a-b) Copy number profile plots with various chromosomal gains in and losses. Note the focal MYCN and CDK4 amplification in case 2. (c) t-SNE of the entire reference cohort, described in [2] confirms the affiliation of the two cases to the respective DNA methylation classes: glioblastoma, IDH wildtype, H3.3 G34 mutant and glioblastoma, IDH wild type, subclass MYCN.
